# Stanford type B aortic dissection is more frequently associated with coronary artery atherosclerosis than type A

**DOI:** 10.1186/s13019-018-0765-y

**Published:** 2018-06-27

**Authors:** Naoki Hashiyama, Motohiko Goda, Keiji Uchida, Yukihisa Isomatsu, Shinichi Suzuki, Makoto Mo, Takahiro Nishida, Munetaka Masuda

**Affiliations:** 1 Department of Cardiovascular Surgery, Yokohama Minami-kyosai Hospital, Mutsuurahigashi 1-21-1, Kanazawa-ku, Yokohama, 236-0037 Japan; 2 Department of Cardiovascular Surgery, Yokohama City University Hospital, Fukuura 3-9, Kanazawaku, Yokohama, 236-0004 Japan; 3 Cardiovascular Center, Yokohama City University Medical Center, Yokohama, Japan; 4 Department of Cardiovascular Surgery, Yokohama Citizen’s Municipal Hospital, Yokohama, Japan

**Keywords:** Acute aortic dissection, Stanford classification, Coronary artery, Athroscrelosis, Coronary angiography

## Abstract

**Background:**

The relationship between aortic dissection and coronary artery disease is not clear. The purpose of this study was to clarify the difference in the rate of coronary artery atherosclerosis between Stanford type A and type B aortic dissection by reviewing our institutional database.

**Methods:**

One hundred and forty-five patients (78 males, 67 females; mean age: 60 ± 12 years) admitted to our hospital with acute aortic dissection who underwent coronary angiography during hospitalization from 2000 through 2002 were enrolled in this study. The background characteristics, coronary risk factors, and coronary angiography findings (number of significant stenoses, stenoses according to Bogaty standards, extent index) of patients were compared between type A (Group A; *n* = 71) and type B dissection (Group B; *N* = 74).

**Results:**

Significantly more patients had prior histories of complications from ischemic heart disease in Group B than in Group A (*P* = 0.04), with no significant differences in comparison to other risk factors observed except for hypertension. Significantly (*p* = 0.005) more stenoses were observed in Group B (1.54 ± 0.04) than in Group A (0.38 ± 0.1). A significantly higher (*P* < 0.05) index score indicating the severity of coronary atherosclerosis was observed in Group B (1.49 ± 0.09) than in Group A (0.72 ± 0.07).

**Conclusions:**

Stanford type B acute aortic dissection was significantly more frequently associated with coronary artery atherosclerosis than type A.

## Background

While the survival rate of surgical patients with acute aortic dissection (AAD) has been improving recently, it remains over 10% in Asian and Western developed countries [1–3]. We previously reported that the ST-T abnormality on admission electrocardiograms in AAD patients was a significant risk factor of in-hospital mortality [4, 5]. The prevalence of coronary artery disease due to not only dissection involved but also atherosclerotic stenosis must therefore be taken into account in the treatment of AAD. However, the relationship between coronary artery diseases and AAD remains unclear.

Recently, coronary artery computed tomography (CT) has been used widely because of its convenience and low invasiveness [6]. However, an angiographic evaluation of coronary arteries is still clinically important and reliable because coronary CT cannot evaluate coronary lesions adequately in cases of severe coronary artery calcification.

The purpose of this study was to clarify the relationship between coronary artery disease and AAD in accordance with Stanford type A and B based on coronary angiogrphy (CAG) by reviewing our local AAD database during a time when we routinely performed CAG in AAD patients.

## Methods

### Study design and patient population

This retrospective cohort study was performed at a single Japanese center in human subjects and was reviewed and approved by the Institutional Review Board at Yokohama City University Medical Center. Informed consent for this study was waived because no individual patients were identified.

We identified 145 patients (78 males, 67 females; mean age: 60 ± 12 years) who had undergone coronary angiography, among 221 patients hospitalized at our center from January 2000 to December 2002, when we routinely performed CAG in AAD patients. Seventy-six patients in whom CAG was not performed were excluded (49 with type A and 27 with type B). Patients presenting with traumatic aortic dissection or Marfan syndrome were also excluded. The remaining 145 patients who underwent coronary angiography were included in this study and divided into 2 groups based on the type of AAD: Stanford type A acute aortic dissection (A; *n* = 71) and Stanford type B (Group B; *n* = 74) aortic dissection.

### Clinical evaluation

The baseline clinical data of the enrolled patients, including the patient demographics, medical history, clinical findings at presentation, imaging study results and preoperative complications, were collected by a retrospective chart review. Type A aortic dissection was defined as observing an intimal flap separating 2 lumina in the ascending aorta with a presentation within 14 days of its onset [7].

### Angiographic analyses of the coronary artery (Fig. 1)

We performed CAG within three weeks after emergency aortic surgery in Group A or admission in Group B. The angiograms were comprehensively analyzed based on the descriptive concepts developed and described by Bogaty et al.: severity, stenosis and extent [8]. In brief, ‘severity’ was defined as the number of major epicardial vessels with stenosis ≥ 75%. A left main trunk with stenosis ≥ 50% was defined as 2 vessels. ‘Stenoses’ were defined as the total number of stenosis ≥ 50%. The *‘*extent’ of atherosclerotic stenosis was quantified by a score as follows: 0, normal: 1, the length of the atherosclerotic stenosis was ≤ 10% of each classified coronary artery: 2, the length was between 10 and 50%: and 3, the length was ≥ 50%. The extent index was the extent score divided by the number of coronary arteries with a proper antegrade flow, allowing a range from 0 to 3 (extent score 0–45 divide by the classical 15 segments of the coronary artery).

**Fig. 1:**
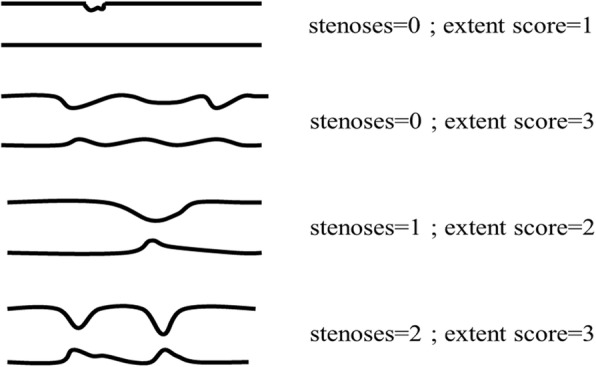
Schematic illustration of the stenoses and extent score as applied to one coronary artery segment. Stenoses are the total number of the coronary lesions narrowing ≥ 50%. The ‘extent’ of atherosclerotic stenosis was quantified by a score as follows: 0, normal with no abnormality; 1, the length of the atherosclerotic stenosis was < 10% of each classified coronary artery; 2, the length was between 10 and 50%; and 3, the length was > 50%

### Statistical analyses

All statistical analyses were performed using the IBM SPSS statics software program, ver. 20 (IBM Corporation, Armonk, NY, USA). All continuous variables are presented as the means ± standard error. Fisher’s exact test and Student’s *t*-tests were used for the univariate analyses. Categorical variables are expressed as absolute numbers or percentages and were compared using chi-squared testing. *P* < 0.05 was considered to be significant.

## Results

### Patient’s characteristics and coronary risk factors

All variables are listed in Tables 1 and 2. The proportion of women was significantly higher in Group A than in Group B (56.3% vs. 36.5%; *p* = 0.02). Ischemic heart disease (3% vs 12%; *P* = 0.04) and arterial hypertension (55% vs. 80%; *p* = 0.003) were observed significantly more frequently in Group B than in Group A. No significant differences were observed between the two groups in the age, height, body weight or other coronary risk factors, including hyperlipidemia, diabetes mellitus, smoking habit and a family history of coronary disease.

**Table 1:** Clinical characteristics of the patients

Variables	Group A	Group B	*p* value
	(*n* = 71)	(*n* = 74)	
Gender (Men/Women)	31/40	47/27	0.02
Age (years)	60 ± 1.4	60 ± 1.3	0.725
Height (cm)	160 ± 1.2	163 ± 0.9	0.052
Weight	62 ± 1.7	65 ± 2	0.21
History of coronary artery disease	2 (3%)	9 (12%)	0.042
Angina pectoris	1	6	
Myocardial infarction	0	1	
Previous coronary surgery	1	2	

A: Stanford type A dissection; B: Stanford type B dissection

Continuous variables are expressed as the mean ± standard error and categorical variables as the number (%) or as indicated

**Table 2:** Coronary risk factors

Variables	Group A	Group B	*p* value
	(*n* = 71)	(*n* = 74)	
Hypertension	39 (55%)	59 (80%)	0.003
Hyperlipidemia	11 (15%)	15 (20%)	0.594
Diabetes Mellitus	5 (7%)	11 (15%)	0.216
Smoking habit	30 (42%)	37(50%)	0.406
Family history	28 (39%)	30 (41%)	0.865

A: Stanford type A dissection; B: Stanford type B dissection

Continuous variables are expressed as the mean ± standard error and categorical variables as the number (%) or as indicated

### Coronary artery analyses

**Fig. 2:**
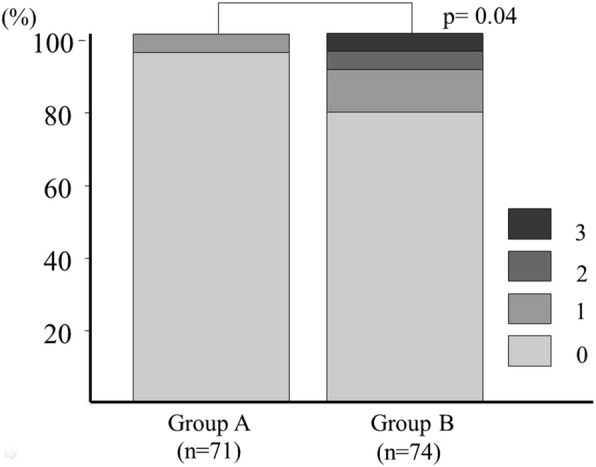
Number of coronary artery diseases defined as ≥ 75% stenosis. Significantly fewer coronary artery diseases were observed in Group A than in Group B (*P* = 0.05)

- Severity:

Only 3 patients in Group A (4.2%) had a single-vessel coronary disease detected by CAG, while 16 patients in Group B (21.6%) had significant coronary disease, including 8 patients with single-vessel disease, 4 with double-vessel disease and 4 with triple-vessel disease. (*p* = 0.04: Fig. 2).

- Stenoses

**Fig. 3:**
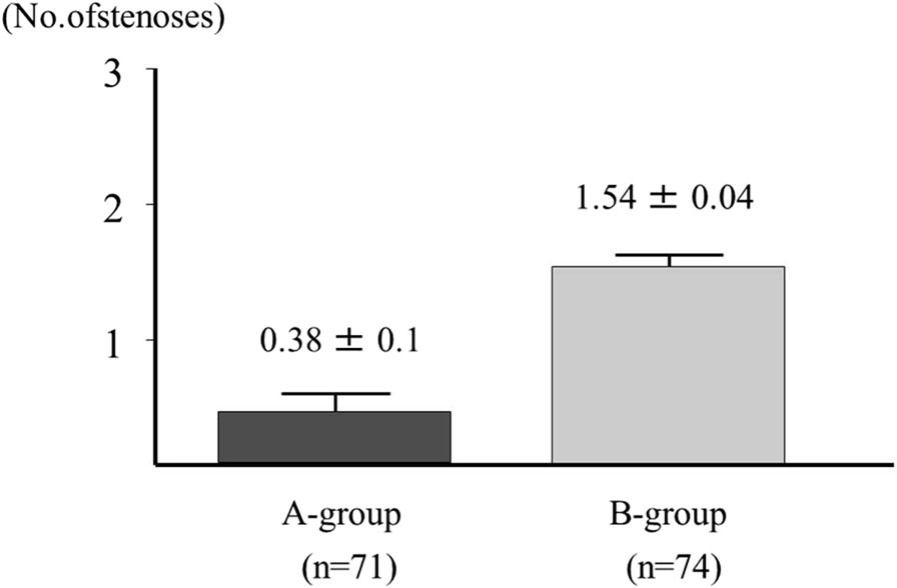
Number of significant stenoses (≥ 50%). Significantly fewer (*p* = 0.005) total numbers of stenoses (≥ 50%) were observed in Group A (26 lesions, 0.38 lesions per patient) than in Group B (114 lesions, 1.54 lesions per patient)

Significantly fewer (*p* = 0.005) total numbers of stenoses (≥ 50%) were observed in Group A (26 lesions, 0.38 lesions per patient) than in Group B (114 lesions, 1.54 lesions per patient: Fig. 3).

- Extent

**Fig. 4:**
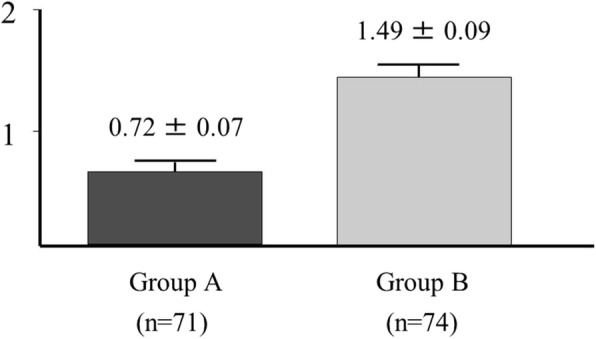
Extent of coronary lesions. There was also a significant difference in the extent index, representing the longitudinal extension of atherosclerotic stenosis between Group A (0.72) and Group B (1.49, *p* = 0.005)

There was also a significant difference in the extent index, representing the longitudinal extension of atherosclerotic stenosis between Group A (0.72) and Group B (1.49, *p* = 0.005: Fig. 4).

## Discussion

AAD naturally has an extremely poor prognosis, and it has been noted that many cases prove fatal shortly after the onset with emergency operation normally required to rescue patients suffering from type A aortic dissection [1–3]. Exclusion of the entry by replacing the dissected ascending aorta and/or aortic arch to the artificial graft is the standard technique for Stanford A dissection; however, surgeons generally do not recognize asymptomatic coronary artery disease without ST-T changes in the electrocardiogram because CAG or coronary CT are not routinely performed before emergent operation. Therefore, treating AAD still carries a potential risk for severe coronary stenosis leading to perioperative myocardial infarction [9, 10].

Only a few reports exist concerning cases of AAD complicated by organic coronary artery atherosclerosis, but in general, complications due to coronary atherosclerosis are still believed to occur with a low frequency [11–13]. Furthermore, there has never been a report describing the relationship between significant atherosclerotic coronary artery stenosis and the Stanford classification. To prevent perioperative myocardial infarction, it is desirable to confirm the presence or absence of significant coronary stenosis during treatment for AAD, especially before emergent operation for type A dissection. Because it is not always possible to examine coronary arteries before emergent operation, it is important to clarify the underlying relationship between atherosclerotic coronary artery stenosis and AAD. Therefore, we examined AAD cases associated with coronary artery atherosclerosis based on coronary angiographical analyses to clarify the incidence of atherosclerotic coronary artery stenosis in these patients.

The present results showed that the number of significant stenoses, overall number of stenoses and extent index indicating the severity of coronary lesions were significantly higher in Group B than in Group A and only 3 of the 71 patients in Group A (4.2%) had single-vessel coronary disease with no patients undergoing CABG in this group. These data suggest that it is not always neccessary to perform a precise coronary artery examination befroe emergent operation for type A dissection, especially in cases with no significant ST-T changes before surgery. In contrast, 16 of the 74 patients in Group B (22%) had coronary artery disease. Thus, in cases of type B dissection, CAG is required to rule out coronary artery disease after the medical treatment of type B dissection.

Why significant differences in the coronary artery lesions were observed between type A and B dissection in our current study in unclear. Some studies have reported that atherosclerosis does not significantly contribute to AAD development [14–16]. However, recent reports from IRAD investigators [17, 18] have shown that the prevalence of coexisting atherosclerosis, aortic aneurysm and even hypertension was significantly higher in type B dissection than in type A dissection, which certainly supports the results of this current study. Indeed, Group B had more atherosclerotic risk factors than Group A in the present study, including proportions of male gender, hypertension and a history of coronary disease. Based on these present and previous findings, we hypothesize that the development of type A dissection is less attributable to atherosclerosis than type B dissection, leading to fewer atherosclerotic coronary lesions.

In addition, several striking reports have described a negative association between atherosclerosis and type A aortic dissection [19], ascending aortic aneurysm [20] and thoracic aortic aneurysm [21]. Although the mechanisms underlying these observations were unclear, those authors hypothesized that the same genetic mutations were responsible for (1) the progressive loss and destruction of elastic fibers or smooth muscle cells in the ascending aortic media leading to aortic dissection and (2) a protective effect against atherosclerosis.

## Conclusions

Stanford type B acute aortic dissection was significantly more frequently associated with coronary artery atherosclerosis than type A. These data suggest that a precise coronary examination is not required before emergent operation for type A dissection, while CAG or coronary CT is required to rule out coronary artery disease in patients with type B dissection.

### Study limitation

This study was based on old data derived from a single center. Furthermore, the small number of subjects did not give the study adequate power to reach robust conclusions.

## Abbreviations

AAD: Acute aortic dissection
CAG: Coronary angiography
CT: Computed tomography
